# Monitoring Wheat Powdery Mildew Based on Hyperspectral, Thermal Infrared, and RGB Image Data Fusion

**DOI:** 10.3390/s22010031

**Published:** 2021-12-22

**Authors:** Ziheng Feng, Li Song, Jianzhao Duan, Li He, Yanyan Zhang, Yongkang Wei, Wei Feng

**Affiliations:** 1 State Key Laboratory of Wheat and Maize Crop Science, Agronomy College, Henan Agriculture University, Zhengzhou 450046, China; fengziheng@stu.henau.edu.cn (Z.F.); songli2021@stu.henau.edu.cn (L.S.); djz2020@stu.henau.edu.cn (J.D.); xmzxhl@henau.edu.cn (L.H.); Yanyan2021@stu.henau.edu.cn (Y.Z.); Weiyongkang@stu.henau.edu.cn (Y.W.); 2 Information and Management Science College, Henan Agricultural University, Zhengzhou 450046, China

**Keywords:** wheat powdery mildew, machine learning, information fusion, remote sensing monitoring

## Abstract

Powdery mildew severely affects wheat growth and yield; therefore, its effective monitoring is essential for the prevention and control of the disease and global food security. In the present study, a spectroradiometer and thermal infrared cameras were used to obtain hyperspectral signature and thermal infrared images data, and thermal infrared temperature parameters (TP) and texture features (TF) were extracted from the thermal infrared images and RGB images of wheat with powdery mildew, during the wheat flowering and filling periods. Based on the ten vegetation indices from the hyperspectral data (VI), TF and TP were integrated, and partial least square regression, random forest regression (RFR), and support vector machine regression (SVR) algorithms were used to construct a prediction model for a wheat powdery mildew disease index. According to the results, the prediction accuracy of RFR was higher than in other models, under both single data source modeling and multi-source data modeling; among the three data sources, VI was the most suitable for powdery mildew monitoring, followed by TP, and finally TF. The RFR model had stable performance in multi-source data fusion modeling (VI&TP&TF), and had the optimal estimation performance with 0.872 and 0.862 of R^2^ for calibration and validation, respectively. The application of multi-source data collaborative modeling could improve the accuracy of remote sensing monitoring of wheat powdery mildew, and facilitate the achievement of high-precision remote sensing monitoring of crop disease status.

## 1. Introduction

In recent years, multiple crop diseases and insect pests have emerged, with considerable impacts on yield and productivity following local outbreaks. According to the United Nations Food and Agriculture Organization (FAO), 20%–40% of crops globally are damaged by disease and insect pests annually [1]. Powdery mildew is the major wheat disease; it causes considerable yield reductions or even no harvest, posing a major threat to wheat production and global food security. Conventional methods of monitoring wheat disease are time-consuming and laborious, and are associated with mechanical damage to crops. Therefore, it is essential to identify and develop approaches of carrying out rapid and damage-free wheat disease monitoring.

Plant disease and insect pest infestations lead to biomass reductions, leaf structure destruction, and chlorophyll and water content reductions. Shifts in chlorophyll, water, and other biochemical components in plant tissues would inevitably yield diverse absorption and reflectance characteristics on the plant reflectance spectrum curve, which provides a theoretical basis and facilitates the real-time monitoring of wheat diseases using remote sensing technologies [2]. In recent years, with continuous advancements in remote sensing technologies, numerous scholars have applied technologies to monitor wheat diseases. Generally, different crops, varieties, and diseases exhibit diverse spectral characteristics, which leads to varying reflectance sensitivities at different bands following disease infestation [3]. Consequently, the identification of crop diseases and crop disease incidence estimation can be achieved based on changes in spectral responses and reflectance characteristics [4,5,6]. Researchers have previously developed disease monitoring indices following the extraction of disease-sensitive bands for monitoring the infestation of crops by bacterial diseases, such as powdery mildew index (PMI) [7], double green vegetation index [8], and red edge vegetation stress index (RVSI) [9].

The modeling algorithms applied in remote sensing influence the accuracy of remote sensing technologies. Today, the algorithms applied in disease and pest monitoring with remote sensing technologies are mainly empirical models and machine learning algorithms [10,11]. Among them, the empirical models are relatively simple; however, the data are easily influenced by external conditions and have poor universality. In recent years, machine learning methods have emerged, with rapid development. Crop disease monitoring models established based on machine learning methods consider training error and generalization ability, and address the challenges associated with slight changes in reflection coefficient during crop disease detection [12,13]. Gu et al. [14] used hyperspectral imaging technologies to monitor tobacco infected by tomato spotted wilt virus and reported that the combination of a successive projections algorithm (SPA) and boosted regression tree was the optimal modeling approach. In addition, Liu et al. [15] established a wheat wilt monitoring model using an improved backward propagation neural network. Wheat is a crop planted in dense rows; if only a single spectral data type is applied in disease monitoring activities, the model is often insensitive to changes in canopy spectrum reflectance, and the reflectance data can be saturated, leading to significant model prediction errors [16,17].

Texture information obtained using imaging spectroscopy tools can reflect disease spot sizes and infestation levels of bacterial diseases [18], in addition to integrating plant morphology and canopy structure information in the spectral data, which enhances the accuracy of remote sensing tools in crop disease monitoring activities [19]. Many researchers have exploited the complementary advantages of spectrum and texture information, which has significantly improved crop growth parameters and the inversion effect of disease severity [20,21]. For example, Guo et al. [22] used vegetation index (VI) and texture features (TF) data obtained using an unmanned aerial vehicle (UAV) platform to establish a wheat stripe rust monitoring model based on partial least squares regression (PLSR). TF can provide plant morphology data that could be applied in the monitoring of crop growth based on remote sensing technologies, which can address the saturation and low accuracy shortcomings associated with single spectral information source-based monitoring, and in turn enhance the robustness of a model and model inversion performance.

Infrared thermal imaging technologies have high sensitivity and early warning capacity. Wheat plants are infected by powdery mildew fungus, and the early symptoms are mostly manifested by changes in internal physiological reactions. Thermal infrared images can reveal temperature changes infected regions that cannot be discerned by visible light images [23]. Mahlein et al. [24] used an infrared thermal instrument (IRT) to measure wheat canopy temperature (CT) and found that the temperature of diseased spikelets was significantly higher than that of healthy spikelets. Therefore, infrared thermal imaging technologies can be used to monitor crop stress during growth and physiological conditions. Many researchers have begun to combine IRT with other remote sensing data sources in plant disease monitoring activities. For example, Zarco-Tejada et al. [25] confirmed that the combination of VI, sun induced fluorescence (SIF), and crop water stress index (CWSI) could be used to effectively monitor diseased trees, and the identification accuracy rate exceeds 80%. In addition, Poblete et al. [26] combined the spectrum, SIF and CWSI, which could effectively distinguish diseased and non-diseased olive trees, whereas Zhang et al. [27] used UAV multi-spectral VI in combination with CT information to estimate disease severity in disease-stressed chickpea, with significantly enhanced detection accuracy. The results of the above studies indicate that thermal infrared data can reliably reflect abnormal conditions in the CT of stressed crops, and can facilitate disease identification and disease classification when combined with other remote sensing data sources.

In the wake of rapid advancements in modern electronic information science, numerous sensors are available for application in the detection of crop morphology and canopy structure, such as reflectance spectrometers, chlorophyll fluorescence meters, and IRT and RGB cameras, which detect crop morphology and growth status based on different factors and principles [28]. However, crop information associated with a single information source is often potentially biased and has certain limitations. Data from different sensor types can be deployed synergistically to enhance target detection and recognition capabilities [29]. Compared to a single sensor data source, multi-sensor data sources can enhance the reliability and robustness of real-time detection [30]. At present, few studies have reported on the monitoring of wheat powdery mildew disease based on a synergy of spectral data and thermal infrared temperature data; in particular, there is a dearth of studies on disease monitoring using approaches that synergize VI, TF and temperature parameters (TP).

To further explore the synergistic effects of multimodal data obtained from different sensors in disease monitoring, in the present study, multimodal data on the incidence of wheat powdery mildew was obtained using hyperspectral surface spectrometer and thermal infrared camera, and compared with ground disease investigations. Multimodal data were obtained using modern modeling and inversion algorithms, such as PLSR, support vector machine regression (SVR), and random forest regression (RFR). The results of the present study could provide a technical basis for the rapid and large-scale monitoring of wheat powdery mildew, and facilitate the prevention and precise control of wheat powdery mildew, in addition to the improvement of pesticide efficiency and food safety.

## 2. Materials and Methods

### 2.1. Experimental Design

Experiment 1 (EXP.1): The experiment was conducted in the 2020–2021 wheat growing season at the Science and Education Demonstration Park (34°51′ N, 113°35′ E) of Henan Agricultural University, Zhengzhou, China. The tested varieties were varieties susceptible to wheat powdery mildew: Aikang 58 and Yumai 49–198. The first crop was corn, and the stalks were crushed and returned to the field. The soil was loam, the 0~30-cm soil contained 0.99–1.18 g kg^−1^ of total nitrogen (N), 0.023–0.034 g kg^−1^ of available phosphorus, 0.114–0.116 g kg^−1^ of available potassium, and 11.4–15.3 g kg^−1^ of organic matter. In the experiments, relatively high water and N fertilizer amounts were used to create favorable conditions for powdery mildew. The amount of N applied was 270 kg·hm^−2^, and the irrigation amount during the wintering period-jointing stage was 900 m^3^·hm^−2^. Powdery mildew fungus was inoculated at the jointing stage, and the wheat was infected from the flowering stage, and the canopy spectrum data were obtained at the flowering and filling stages. Other field management approaches were similar to those applied locally.

Experiment 2 (EXP.2): Carried out simultaneously with experiment 1, experiment 2 was a variety comparison experiment in the field, involving Yanzhan 4110, Nongmai 18, Zhoumai 27, Jinfeng 205, Zhengmai 1342, Xumai 318, Bainong 207, and Xinmai 26. The amount of N applied was 225 kg·hm^−2^, and the irrigation amount during the wintering period-jointing stage was 675 m^3^·hm^−2^. The experimental area was close to fences and pig farms, and the terrain was low-lying. Due to the terrain, air humidity, rainfall, and diseases in previous years, the wheat growth environment was suitable for the occurrence and spread of wheat powdery mildew, without field inoculation. Disease emergence was natural and more severe. Other field management approaches were similar to those applied in EXP.1.

### 2.2. Ground Data Collection

#### 2.2.1. Investigation of Powdery Mildew

During the wheat flowering and filling periods, wheat powdery mildew incidence was investigated manually, and 77 and 37 samples were collected in EXP.1 and EXP.2, respectively. About 0.2 m^2^ of the experimental area was investigated at each point, and 20 representative wheat plants were selected to test for powdery mildew infection. The survey was conducted in strict accordance with the technical specifications for crop disease monitoring (Chinese Standard: NY/T 2738.2-2015) [31]. The ratio of the leaf area covered by the diseased mycelium layer on the diseased leaf to the total leaf area was expressed based on a grading method, with eight levels representing 1%, 5%, 10%, 20%, 40%, 60%, 80%, and 100% coverage. The grid method was used to calculate the ratio of the diseased spot area to the leaf area. The operation involved using grids to cover the leaves, recording the total number of grids with disease spots, to facilitate the calculation of the ratio of the diseased spot area to leaf area. The closest value between grades was selected as the actual level. For example, at onset with a severity of less than 1%, the coverage was considered 1%. The average severity of diseased leaves was calculated as follows (1):

(1) $$D=\frac{\sum^{\;}\left(Di\times Li\right)}{L}\times 100$$

where, D is the average disease severity in leaves, and the unit is percentage (%); Di is each severity value; Li is the number of diseased leaves corresponding to each severity value, and the unit is slice; and L is the total number of leaves under investigation, and the unit is slice.

On the basis of the severity of disease in leaves, the disease index (***DI***) is calculated to represent the average level of disease occurrence (Equation (2)).

(2) DI=F×D×100

where, DI is the disease index; F is the diseased leaf rate; D is the average severity of disease in leaves.

#### 2.2.2. Canopy Spectrum Data Measurement

From 10:00 to 14:00 (Beijing local time) with little wind and clear weather, a FieldSpec handheld spectrometer (FieldSpec Handheld 2, Analytical Spectral Devices, Boulder, CO, USA) was used to obtain wheat canopy spectrum data, and the probe was 1.0 m from the top of the wheat crop. The field of view of the spectrometer was 25°, in the 325–1075 nm band, the spectral sampling interval was 1.4 nm, and the spectral resolution was 3.0 nm. A 0.4-m × 0.4-m BaSO_4_ calibration plate was used to calculate black and baseline reflectance. Ten spectral reflectance values were recorded at each sampling point as samples, and the average value was considered the spectral reflectance of the sampling area.

#### 2.2.3. Thermal Infrared Image and RGB Image Acquisition

**Figure 1:**
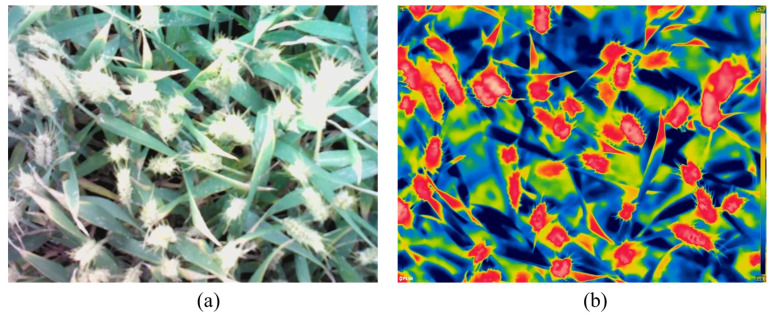
Wheat canopy RGB image (**a**) and thermal infrared image (**b**) obtained using thermal infrared camera.

An FLIR T650sc thermal infrared camera (FLIR Systems, Inc., Wilsonville, OR, USA) was used to obtain the wheat canopy temperature (CT) and RGB images. The device has dual thermal infrared and visible light sensors, and the image resolution is 640 × 480 pixels. Synchronous with the spectral reflectance measurement, the lens was 1.0 m from the top of the wheat crop, and the thermal infrared and RGB images were obtained vertically (Figure 1).

### 2.3. Data Processing Methods

#### 2.3.1. Spectral Vegetation Index (VI)

Before extracting the VIs, the bands with high noise before 400 nm and after 1000 nm were removed, and then the Savitzky-Golay function was used to smoothen the spectra in MATLAB 7.0 (The MathWorks Inc., Natick, MA, USA). VIs associated with the disease were pre-selected by consulting relevant literatures (Table 1). Considering the potential existence of the collinearity problem among VIs, SPA was used to optimize VIs and reduce their multicollinearity. SPA is a forward variable selection method that selects characteristic variables by calculating the sizes of the projection vector of the remaining variables and the selected variables, which can ensure that the linear relationship between the selected variables is minimized, so as to eliminate redundant information between variables and reduce multicollinearity, to achieve the purpose of selecting sensitive variables [32].

#### 2.3.2. RGB Image Texture Features (TF)

**Figure 2:**
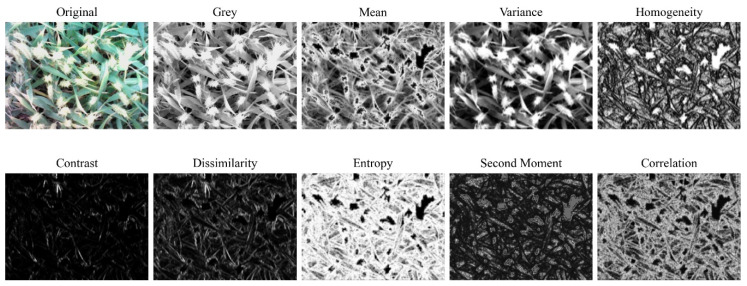
Eight texture feature maps of gray-level co-occurrence matrix from RGB image of wheat canopy in the 0° direction.

**Figure 3:**
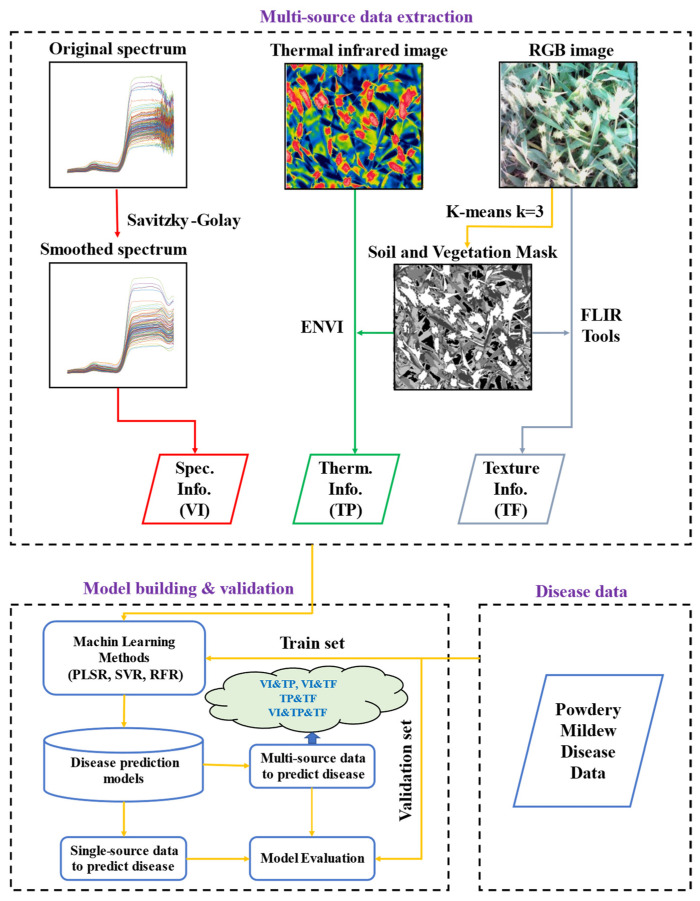
A workflow diagram of feature extraction and modeling.

The gray level co-occurrence matrix (GLCM) method, proposed by Haralick in 1973 [50] is one of the most widely used texture extraction methods. The method has the advantages of rotation invariance, multi-scale characteristics, and low computational complexity, and is widely used in image processing, pattern recognition, and remote sensing monitoring [51,52]. In ENVI (Harris, Bloomfield, CO, USA), the gray level image of the RGB image was subjected to 3 × 3 sliding filtering using GLCM. Eight texture feature maps in the directions of 0°, 45°, 90° and 135° were extracted (Figure 2, Table 2), and the average of four directions was taken as the final texture feature map. To ensure that the extracted texture features are all based on canopy vegetation, a K-means clustering algorithm is used for bare soil rejection. The soil and vegetation mask is shown in Figure 3.

#### 2.3.3. Thermal Infrared Temperature Parameters (TP)

The thermal infrared image was annotated and combined with K-means clustering segmentation results (Figure 3) using FLIR Tools (FLIR Systems Inc., Wilsonville, OR, US), and the temperature parameters were extracted. Considering that CT changes with the daily change in atmospheric temperature, the canopy temperature difference (CTD), canopy temperature ratio (CTR), and normalized relative canopy temperature (NRCT) were extracted to eliminate the influence of atmospheric temperature on CT. The temperature parameter formula was as follows:

(3) $$CTD=CT_{i}-AT$$

(4) $$CTR=\frac{CT_{i}}{AT}$$

(5) $$NRCT=\frac{CT_{i}-CT_{min}}{CT_{max}-CT_{min}}$$

where, ***AT*** is the atmospheric temperature, $CT_{i}$ is the ***CT*** of the i-th pixel in the image, $CT_{max}$ is the highest temperature measured in the entire experimental field, and $CT_{min}$ is the lowest temperature measured in the entire experimental field.

#### 2.3.4. Estimation Model

(1) PLSR

PLSR is a classic modeling method, which includes the characteristics of principal component analysis (PCA), canonical correlation analysis, and multiple linear regression analysis, and is often used for quantitative analysis in remote sensing [53]. PLSR transforms the original variables with high data redundancy into a few variables by selecting the optimal latent variables, to describe the linear model of the relationship between the predicted value and the true value.

(2) SVR

The basic idea of SVR is to use training samples to establish a regression hyperplane, and to approximate the samples to the hyperplane to minimize the total deviation from the sample point to the plane [54]. The commonly used kernel functions of the SVR algorithm include the linear kernel function, radial basis function (RBF) kernel function, polynomial kernel function, and Sigmoid kernel function. Among them, the RBF kernel function can handle the complex nonlinear problem between the independent variable and the dependent variable.

(3) RFR

RFR is a machine learning algorithm based on a classification regression tree [55]. RFR uses the bootstrap resampling method to extract multiple samples from the original sample, models each bootstrap sample into a decision tree, combines them into multiple decision trees for prediction, and then applies the majority voting method to determine the final classification result of the joint prediction model. The advantage of the method is that the training speed is relatively fast and it does not require cross-validation. In addition, the randomness of sampling and feature selection make the random forest averts overfitting [56]. It is widely used in classification and prediction in remote sensing-based monitoring activities.

#### 2.3.5. Model Validation

With VI, TP and TF as independent variables, and DI as the dependent variable, a monitoring model for wheat powdery mildew disease index was established based on the three algorithms above. The workflow from feature extraction to model building and evaluation was demonstrated in Figure 3. To make the model evaluation results more objective, EXP.1 test data were used as the modeling set, and EXP.2 test data were used as the verification set. The accuracy of the wheat powdery mildew disease index monitoring model was evaluated based on three indicators: coefficient of determination (R^2^), root mean square error (RMSE), and relative error (RE). The closer ***R***^2^ is to 1, the lower the ***RMSE***, and the lower the ***RE***, the higher the accuracy of the monitoring model. The formula was as follows:

(6) $$R^{2}=\frac{\sum_{i=1}^{n}{\left(x_{i}-\bar{x}\right)}^{2}\times {\left(y_{i}-\bar{y}\right)}^{2}}{\sum_{i=1}^{n}{\left(x_{i}-\bar{x}\right)}^{2}\times \sum_{i=1}^{n}{\left(y_{i}-\bar{y}\right)}^{2}}$$

(7) $$RMSE=\sqrt{\frac{\sum_{i=1}^{n}{\left(y_{i}-x_{i}\right)}^{2}}{n}}$$

(8) $$RE\;\left(\%\right)=\sqrt{\frac{1}{n}\times \overset{n}{\underset{i=1}{\sum }}{\left(\frac{y_{i}-x_{i}}{x_{i}}\right)}^{2}}\times 100$$

where, $x_{i}$, $\bar{x}$, $y_{i}$, and $\bar{y}$ are the measured DI, average DI, predicted DI, and average DI, respectively; n is the number of samples.

## 3. Results

### 3.1. Changes in Wheat Canopy Spectra under Different Powdery Mildew Severity Levels

**Figure 4:**
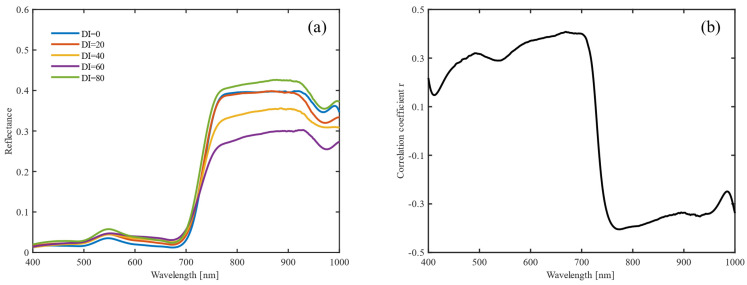
Spectral reflectance changes (**a**) of wheat canopy and its correlation (**b**) with disease index.

With an increase in DI, the spectral reflectance of the visible light band from 400 to 780 nm increased gradually, and the discrimination of DI was better (Figure 4a). The spectral reflectance of the near-infrared bands region across 780 nm–1000 nm was less distinguishable when the disease was mild; when the disease was more than moderate, the spectral reflectance increased gradually; and when the disease was severe (such as DI = 80), the spectral reflectance rose sharply due to severe damage to the canopy structure, even higher than the spectral reflectance of healthy wheat. From the perspective of the correlation between disease severity and reflectance (Figure 4b), there was a positive correlation in the visible light band from 400 to 730 nm, and a negative correlation in the near-infrared region from 730 to 1000 nm, especially at 600–700 nm (r = 0.373–0.431, probability value, *p* < 0.01) and 780–960 nm (r=−0.355–−0.294, *p* < 0.01), which can be considered as disease-sensitive bands for the real-time monitoring of disease progression.

### 3.2. Selection of Vegetation Index

**Figure 5:**
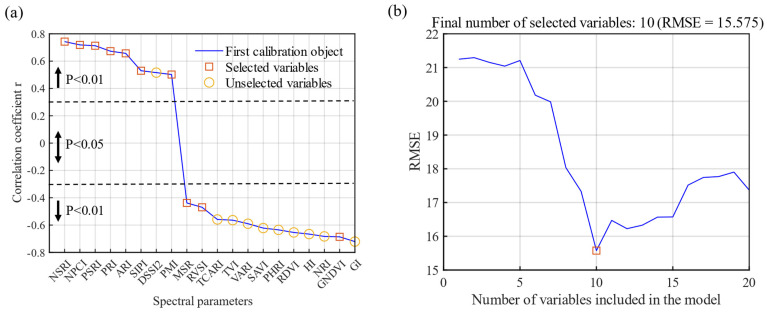
Root mean square error (**a**) in the optimal variables selected using successive projections algorithm (SPA) and correlation (**b**) between vegetation index (VI) and disease index (DI).

**Figure 6:**
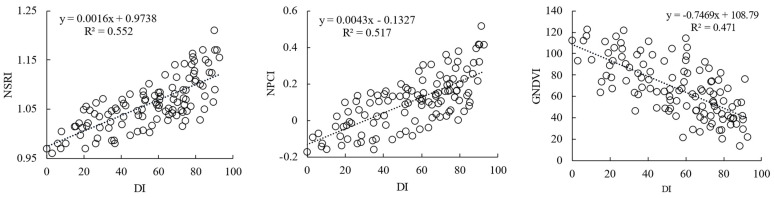
Linear relationship of the optimal vegetation indices with wheat disease index (DI).

Based on the reported VIs related to plant disease, the correlations between 20 VIs and DI were analyzed. The VI with the highest correlation was NSRI (r = 0.743) (Figure 5a), followed by GI and NPCI. Because VI is a combination of bands, and there is a certain degree of information duplication between bands, there is considerable multicollinearity among the spectral parameters. Therefore, the SPA algorithm is used to optimize the VI. The minimum number of sensitive variables extracted was two, and the maximum number of sensitive variables was twenty. RMSE decreased with an increase in the number of variables. RMSE was the minimum (RMSE = 15.575) when the number of variables was 10; however, with an increase in the number of variables, the RMSE increased gradually (Figure 5b). After SPA screening, there were 10 VIs, namely NSRI, NPCI, PSRI, PRI, ARI, SIPI, PMI, MSR, RVSI, and GNDVI. According to the results, when a single VI was used to estimate the DI, the linear R^2^ was low (R^2^ < 0.56), and the error in monitoring powdery mildew disease was relatively large (Figure 6).

### 3.3. Selection of Texture Feature Parameters

**Figure 7:**
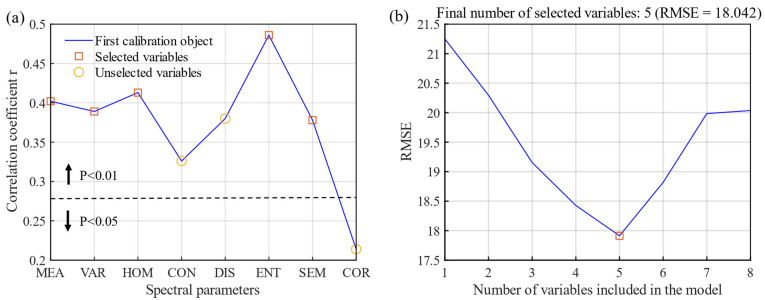
Correlation (**a**) between texture feature parameter and disease index (DI) and the root mean square error (**b**) for the optimal variables selected using SPA.

Analysis of the correlation between TF parameters of canopy RGB images and DI showed that the correlation coefficients were all positive. Excluding the correlation coefficient between the correlation and DI, which was not significant, all the others were significant, among which entropy was the highest (r = 0.486, *p* < 0.01) (Figure 7a). The eight extracted TFs were all calculated from grayscale images, and considering the potential multicollinearity among TFs, the SPA algorithm was used to optimize the variables. The RMSE was the lowest (RMSE = 18.043) when the number of TF variables was five. Five TF parameters, mean, variance, homogeneity, entropy, and second moment, were selected as the input variables in the estimation model (Figure 7b).

### 3.4. Selection of Thermal Infrared Temperature Parameters

**Figure 8:**
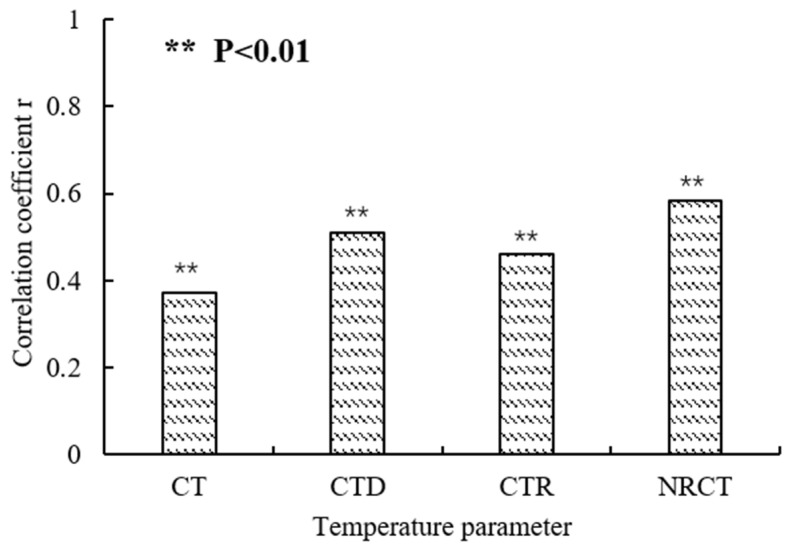
Correlation coefficient between temperature parameters and DI.

The TP parameters from thermal infrared images were extracted to analyze their correlation with DI, and the results showed that the correlation coefficient between CT and DI was 0.382, and was significant (*p* < 0.01) (Figure 8). Considering CT changes with daily atmospheric temperature changes, the CTD, CTR and NRCT were extracted to eliminate the influence of atmospheric temperature on CT. After eliminating the influence of atmospheric temperature, the significant level of correlation of TP was further improved, with a very significant level (*p* < 0.01) observed. Combining the principle of strong correlation and eliminating duplicate information, two TPs, CTD, and NRCT, were selected as input variables for the subsequent steps of modeling and analysis.

### 3.5. Comparison of Different Model Algorithms Based on Single Data Sources

**Figure 9:**
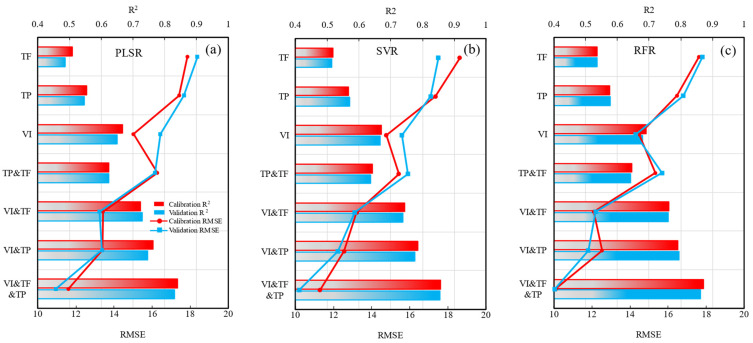
Prediction performance of different models with various input feature types.

With a single data source as an independent variable, three methods, including, PLSR, SVR and RFR, were used to invert the DI of wheat powdery mildew (Table 3, Figure 9). Comprehensive comparison revealed that the RFR model performed optimally, followed by the SVR and PLSR models. Based on the performance results of the three data sources, regardless of which method was used to estimate wheat DI, the performance of the VI was the best, followed by TP and TF. Based on combinations modeling methods and independent variable data type, the RFR method with VI as the independent variable was the best combination, with R^2^, RMSE, and RE values of 0.690, 14.488, and 18.42%, respectively, in the calibration set, and R^2^, RMSE, and RE values of 0.680, 14.298, and 18.16%, respectively, in the validation set. The SVR method with VI as an independent variable was the second best combination, with R^2^, RMSE and RE values of 0.670, 14.757, and 18.69%, respectively, in the calibration set, and R^2^, RMSE, and RE values of 0.666, 15.578, and 18.16%, respectively, in the validation set.

### 3.6. Comparison of Different Model Algorithms Based on Multi-Source Data Combination

To fully exploit the information obtained from different data sources, TP, TF, and VI were combined to carry out a comparative analysis of three modeling methods (Table 4, Figure 9). After fusing TF on the basis of VI data, the average R^2^, RMSE, and RE values in the calibration set were 0.743, 12.91, and 17.57%, respectively, with the R^2^ value representing an average increase of 10% when compared with the R^2^ of single VI data source. The mean R^2^, RMSE, and RE values in the validation set were 0.742, 12.849, and 17.71%, respectively, and the R^2^ value represented a 11.6% increase when compared to the R^2^ of the single VI data source. The addition of TP based on VI data enhanced the accuracy of the combined model. The average R^2^ values in the validation and calibration sets were 0.779 and 0.772, respectively, the RMSE values were 12.823 and 12.467, respectively, and the RE values were 15.56% and 15.62%, respectively. The R^2^ values were 15.4% and 16% higher, respectively, than the R^2^ values based on the single VI data source. In addition, in the combined TP and TF modelling, the estimation performance was superior to those of the single data sources, both TP or TF; however, the model was inferior to VI based on both the calibration and validation sets. The results indicate that when using single data source-based VI as a benchmark, TF has a minor positive effect on model accuracy when performing multi-data collaboration modeling, whereas TP has a relatively high positive effect on model improvement.

**Figure 10:**
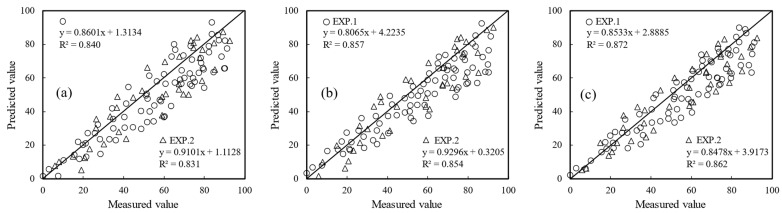
Comparison of three data source fusion models based on (**a**) PLSR, (**b**) SVR and (**c**) RFR algorithm.

Three modeling methods were used to synergize three data sources, VI, TF, and TP. The R^2^ values of the calibration and validation sets were further improved compared with the R^2^ value following combination of the two data sources. The average R^2^ values of the calibration and validation sets in the model with the three data sources combined were 0.856 and 0.849, respectively, the RMSE values were 10.997 and 10.399, respectively, and the RE values were 13.42% and 13.16%, respectively. The R^2^ values were 26.8% and 27.6% higher, respectively, than the R^2^ values of the single VI data source model. Comparison of the modeling algorithm results showed that the RFR model had the highest R^2^, the lowest RMSE and RE, and the greatest DI predictive capacity, followed by the SVR model and PLSR models (Figure 10). The R^2^ values of the RFR fusion model of the three data sources were 0.872 and 0.862 in the calibration and validation sets, respectively, the RMSE values were 10.108 and 10.049, respectively, and the RE values were 12.54% and 12.31%, respectively. The above results indicate that collaborative modeling with multiple data sources is superior to single data source-based modeling, with the combined model exhibiting better fit, accuracy, and predictive ability (Figure 9).

## 4. Discussion

### 4.1. Combining VI and TF to Monitor Crop Diseases

Previous literature has confirmed the importance of reflectance spectrum data in crop disease monitoring and its application prospects. The visible light and near-infrared regions are the sensitive bands for spectral identification of different crop diseases and insect pests; furthermore, the spectral sensitivity bands of different crops and different diseases vary. The sensitive bands of wheat powdery mildew are located at 490–780 nm [57], and wheat powdery mildew monitoring is mainly based on the sensitive band [58,59], and different forms of disease VI can be established according to the reflection characteristics of the disease [7,8,9]. Disease emergence involves gradual development that alters internal tissue physiology and biochemistry, and, in turn, the external morphological structure, and then manifests externally as disease that can be detected by remote sensing. Due to the combined effects of internal and external factors, such as mesophyll cells, water, chlorophyll, and leaf yellowing and dryness, the ability to extract disease information from a single band is often limited. The VIs with good performance in the present study were NSRI, NPCI, and CVI. Among them, NSRI performed optimally, with a linear R^2^ of only 0.552, which hardly meets the information requirements for accurate crop protection.

The onset of powdery mildew disease has a significant bottom-up characteristic. In the early and middle stages of the disease, the disease is mainly concentrated in the middle and lower levels of the plant. However, the canopy reflectance spectra data mainly originate from the upper level, which leads to lack of consistency between the collected canopy spectra data and disease characteristics, and increases the challenge of monitoring powdery mildew using canopy spectrum data only. Therefore, the use of multivariate analysis methods to identify and monitor disease has become a hotspot in quantitative remote sensing research.

In the present study, multiple VIs were used as independent variables, and three algorithms PLSR, SVR, and RFR were used to predict DI. The results showed that the RFR model had the highest monitoring accuracy; however, R^2^ was still lower than 0.7. From the perspective of precise crop protection and disease prevention and control, the spectral data could not be used to monitor wheat powdery mildew reliably. Some scholars have attempted to incorporate fluorescence data in modeling when using hyperspectral data for disease monitoring, and achieved good monitoring results [60]. When a pathogen infects plants, the canopy structure changes following physiological and biochemical responses, and the TF can reflect the change in canopy structure caused by pathogen infestation to a certain degree [61,62]. Researchers have used hyperspectral VI in combination with TF to monitor wheat stripe rust, and reported that the estimation results of the two combined data sources were significantly better than that of the single data source [22]. In the present study, the VI and TF were modeled together, and model accuracy improved when compared with when the VI from a single data source was used. However, the highest accuracy of the combined model in the validation set was only 0.761, the optimization effect was limited, and it did not satisfy the requirements of accurate monitoring, which could be due to the gradual senescence of wheat leaves after the flowering period, and the increased background complexity of withered plants. Furthermore, multiple factors in some plots, such as disease, drought, senescence, and atmospheric temperature, which make it impossible to accurately distinguish whether the withered leaves and structural changes are attributed to disease stress, could have adversely influenced the modelling findings.

### 4.2. Combining VI and TP to Monitor Crop Disease

Thermal infrared imaging technologies have great application potential in remote sensing monitoring activities [63,64,65]. After crops are infected by fungi and pathogens, cell membrane permeability increases, water is lost, and plants exhibit dehydration and wilting. In addition, stomata are closed and heat loss on the leaf surface changes, which leads to leaf surface temperature response. At the onset of crop disease, changes in heat radiation energy caused by plant water loss, stomata closure, and increased respiration can be intuitively reflected in infrared heat maps; however, most studies have focused on disease classification and disease identification. Calderón et al. [66] demonstrated the capacity of using canopy temperature information and hyperspectral VI to identify olive trees with yellow dwarf disease; however, they did not estimate disease severity. In the previous monitoring research on wheat powdery mildew, no studies have reported the combination of VI and TP. The correlation analyses carried out in the present study showed that the thermal infrared temperature was sensitive to disease, and it was more effective to convert CT into CTD, CTR, and NRCT. Compared to canopy TF, temperature information had a greater role in disease monitoring. The RFR model performed best (R^2^ = 0.577) and was slightly more accurate than the TF-RFR model; however, it was significantly less accurate than the VI-RFR model. VI as a single data source was more suitable for monitoring wheat powdery mildew, followed by canopy TP and canopy TF. To further improve the information limitations of single data sources, VI and canopy TP were modeled together (VI&TP). Model accuracies of different algorithms were higher than that of VI&TF on the whole, indicating that canopy temperature information has great application potential in disease monitoring.

Previous studies have also demonstrated that spectral information, texture information, and thermal infrared information have the ability to monitor crop diseases [22,24,26]; however, no study has reported their joint application in wheat powdery mildew monitoring. To that end, the present study conducted fusion modeling based on VI, TF, and TP (VI&TF&TP). According to the results, the combination of the three data sources had obvious advantages over single data sources or two combined data sources. Among them, the R^2^ values of the three data source models based on the RFR algorithm was 0.862, which provides technical support and a reference method for the prevention and precise control of wheat powdery mildew.

### 4.3. Machine Learning Algorithms in Disease Monitoring

In the wake of rapid advancements in computer modeling science, machine learning technology has been applied extensively in crop disease monitoring, with the achievement of remarkable results [67,68]. Jiang et al. [69] demonstrated the high estimation capacity of the RFR model in the monitoring of mangrove disease and insect pests. In addition, Zhang et al. [70] demonstrated the good classification performance of the RFR model in the identification of wheat grains infected with *Fusarium*. In the present study, three modeling methods (PLSR, SVR, RFR) were used to monitor wheat powdery mildew DI. The RFR model performed best, regardless of whether it was based on single data source modeling or multi-source data modeling. This is mainly because the RFR algorithm has good anti-noise ability and does not easily exhibit over-fitting [71]. In the present study, SVR was used to integrate information from three data sources, and the average accuracy that the model achieved was 0.77. Considering the operation efficiency and prediction accuracy of the model, the method is effective for monitoring the disease. In contrast, the performance of the PLSR model was slightly worse, which might be because PLSR was better at addressing multicollinearity between parameters [72], and the parameters used in the present study were optimized by the SPA algorithm, which eliminated the influence of multicollinearity, resulting in an inability to maximize the performance of the PLSR model.

Although the overall performance of the RFR model in the present study was the best, the estimated value was lower than the actual value under more severe disease conditions, which was also observed in the other model algorithms. Generally, the greater the population density of wheat powdery mildew, the worse the air permeability, and the more severe the disease, which decreases the sensitivity of spectral and thermal imaging data to disease severity. Under the condition of multi-source data fusion, the saturation of the model was alleviated, which also demonstrates the effectiveness of multi-data source fusion.

When applying different model algorithms to monitor wheat powdery mildew in collaboration with VI, TF, and TP, the present study did not consider the contribution rates of different data source parameters to the model. How to use different algorithms to determine the weights of different data source parameters to further improve the accuracy of the model remains to be further studied. The occurrence and characteristics of powdery mildew are certainly associated with crop variety, growth period, and other diverse factors. Using targeted information extraction algorithms to clarify the effects and contribution levels of each influencing factor could facilitate the integration multiple effect factors to accurately monitor disease occurrence, and provide a theoretical basis for crop protection and precise operations.

## 5. Conclusions

Based on multi-source data fusion and machine learning, the present study explores the application potential of canopy spectral vegetation index, thermal infrared information, and texture feature information obtained using different sensors in wheat powdery mildew monitoring. In the case of wheat disease index prediction based on single data source, spectral information is better than thermal infrared information and texture features. Regardless of the modeling method, the results obtained following the fusion of data from multiple sources are more reliable than the data obtained from a single data source. When using the combination of vegetation index, thermal infrared information and texture features, higher prediction precision can be achieved. Regardless of whether single data source or multi-source data is used, the monitoring accuracy of the RFR model is higher than that of other algorithm models. Therefore, the combination of multi-source data fusion and the RFR model have broad application prospects in wheat powdery mildew monitoring, which could not only promote disease prevention and control but also reduce pesticide use and enhance the efficiency of disease prevention and control activities. However, the models identified should be tested under different crop types, growth stages, and environmental conditions, to further evaluate the robustness of the models.

## Figures and Tables

**Table 1:** Spectral vegetation indices.

Vegetation Index	Formula	References
Modified simple ration (MSR)	$\mathrm{MSR}=\left(R_{800}/R_{670}-1\right)/{\left(R_{800}/R_{670}+1\right)}^{0.5}$	[33]
Photochemical reflectance index (PRI)	$\mathrm{PRI}=\left(R_{531}-R_{570}\right)/\left(R_{531}+R_{570}\right)$	[34]
Physiological reflectance index (PHRI)	$\mathrm{PhRI}=\left(R_{550}-R_{531}\right)/\left(R_{550}+R_{531}\right)$	[35]
Transformed chlorophyll absorption in reflectance index (TCARI)	$\mathrm{TCARI}=3\left(R_{700}-R_{650}\right)-0.2\left(R_{700}/R_{500}\right)/\left(R_{700}/R_{670}\right)$	[36]
Red-edge vegetation stress index (RVSI)	$\mathrm{RVSI}=\left(\left(R_{712}+R_{752}\right)/2\right)-R_{732}$	[37]
Structural independent pigment index (SIPI)	$\mathrm{SIPI}=\left(R_{800}-R_{445}\right)/\left(R_{800}-R_{680}\right)$	[38]
Visible atmospherically resistant index (VARI)	$\mathrm{VARI}=\left(R_{550}-R_{670}\right)/\left(R_{550}+R_{670}-R_{480}\right)$	[39]
Renormalized difference vegetation index (RDVI)	$\mathrm{RDVI}=\left(R_{800}-R_{670}\right)/{\left(R_{800}+R_{670}\right)}^{0.5}$	[40]
Anthocyanin reflectance index (ARI)	$\mathrm{ARI}={\left(R_{550}\right)}^{-1}/{\left(R_{700}\right)}^{-1}$	[41]
Damage sensitive spectral index 2 (DSSI2)	$\mathrm{DSSI}2=\left(R_{747}-R_{901}-R_{537}-R_{572}\right)/\left(R_{747}-R_{901}+R_{537}-R_{572}\right)$	[42]
Greenness index (GI)	$\mathrm{GI}=R_{554}/R_{677}$	[43]
Plant senescence reflectance index (PSRI)	$\mathrm{PSRI}=\left(R_{680}-R_{500}\right)/R_{750}$	[44]
Normalized pigment chlorophyll Index (NPCI)	$\mathrm{NPCI}=\left(R_{680}-R_{430}\right)/\left(R_{680}-R_{430}\right)$	[43]
Nitrogen reflectance index (NRI)	$\mathrm{NRI}=\left(R_{570}-R_{670}\right)/\left(R_{570}-R_{670}\right)$	[45]
Healthy index (HI)	$\mathrm{HI}=\left(R_{534}-R_{698}\right)/\left(R_{534}+R_{698}\right)-0.5R_{704}$	[7]
Powdery mildew index (PMI)	$\mathrm{PMI}=\left(R_{520}-R_{584}\right)/\left(R_{520}+R_{584}\right)-R_{724}$	[7]
Triangular vegetation index (TVI)	$\mathrm{TVI}=0.5\left[120\left(R_{750}-R_{550}\right)-200\left(R_{670}-R_{550}\right)\right]$	[46]
Green normalized difference vegetation index (GNDVI)	$\mathrm{GNDVI}=\left(R_{800}-R_{550}\right)/\left(R_{800}+R_{550}\right)$	[47]
Nir shoulder region index (NSRI)	$\mathrm{NSRI}=R_{890}/R_{780}$	[48]
Soil-adjusted vegetation index (SAVI)	$\mathrm{SAVI}=1.5\left(R_{800}-R_{670}\right)/\left(R_{800}+R_{670}+0.5\right)$	[49]

**Table 2:** Texture feature calculation formula.

Texture	Equation	Description
Mean, MEA	$\mathrm{MEA}=\overset{G}{\underset{i,j=1}{\sum }}\left(iP\left(i,j\right)\right)$	Reflects the average of the greyscale
Variance, VAR	$\mathrm{VAR}=\overset{G}{\underset{i=1}{\sum }}\overset{G}{\underset{J=1}{\sum }}{\left(i-u\right)}^{2}P\left(i,j\right)$	Reflects the magnitude of grey scale variation
Homogeneity, HOM	$\mathrm{HOM}=\overset{G}{\underset{i=1}{\sum }}\overset{G}{\underset{J=1}{\sum }}\frac{P\left(i,j\right)}{1+{\left(i-j\right)}^{2}}$	Reflects the roughness of image texture
Contrast, CON	$\mathrm{CON}=\overset{G}{\underset{i=1}{\sum }}\overset{G}{\underset{J=1}{\sum }}{\left(i-j\right)}^{2}P\left(i,j\right)$	Reflects the local variations in the gray-level co-occurrence matrix
Dissimilarity, DIS	$\mathrm{DIS}=\overset{G}{\underset{i=1}{\sum }}\overset{G}{\underset{J=1}{\sum }}P\left(i,j\right)\left|i-j\right|$	Same as contrast, used to detect similarity
Entropy, ENT	$\mathrm{ENT}=-\overset{G}{\underset{i=1}{\sum }}\overset{G}{\underset{J=1}{\sum }}P\left(i,j\right)logP\left(i,j\right)$	Reflects the degree of the gray distribution and the thickness of the texture
Second moment, SEM	$\mathrm{SEC}=\overset{G}{\underset{i=1}{\sum }}\overset{G}{\underset{J=1}{\sum }}P^{2}\left(i,j\right)$	Reflects the homogeneity of an image’s distribution of greyscale
Correlation, COR	$\mathrm{COR}=\overset{G}{\underset{i=1}{\sum }}\overset{G}{\underset{J=1}{\sum }}\frac{\left(i-MEA_{j}\right)\left(j-MEA_{j}\right)P\left(i,j\right)}{\sqrt{VAR_{i}}\sqrt{VAR_{j}}}$	Reflects the length of the extension of a certain grey value in a certain direction

Note: i and j indicate the row and column number of the images, respectively; P(i,j) is the relative frequency of two neighboring pixels.

**Table 3:** Estimation performance of single data source model based on different algorithms.

**Independent Variable Type**	**Number of Variables**	**Modeling Method**	**Calibration Set**			**Validation Set**		
			**R^2^**	**RMSE**	**RE**	**R^2^**	**RMSE**	**RE**
VI	10	PLSR	0.666	15.014	19.24%	0.650	16.425	19.86%
		SVR	0.670	14.757	18.69%	0.666	15.578	19.28%
		RFR	0.690	14.488	18.42%	0.680	14.298	18.16%
TF	5	PLSR	0.509	17.852	32.03%	0.486	18.367	32.05%
		SVR	0.517	18.616	30.70%	0.514	17.489	30.23%
		RFR	0.537	17.621	29.18%	0.537	17.799	27.95%
TP	2	PLSR	0.553	17.420	29.27%	0.546	17.673	29.66%
		SVR	0.567	17.347	28.96%	0.571	17.094	27.13%
		RFR	0.575	16.470	27.83%	0.577	16.791	27.79%

**Table 4:** Estimation performance of multi-source collaboration models based on different algorithms.

Independent Variable Type	Number of Variables	Modeling Method	Calibration Set			Validation Set		
			R^2^	RMSE	RE	R^2^	RMSE	RE
TP&TF	7	PLSR	0.624	16.265	21.67%	0.623	16.151	22.67%
		SVR	0.641	15.413	20.39%	0.637	15.900	20.91%
		RFR	0.646	15.328	20.29%	0.641	15.687	20.77%
VI&TF	15	PLSR	0.723	13.417	18.37%	0.728	13.236	18.15%
		SVR	0.744	13.211	17.33%	0.738	13.107	17.27%
		RFR	0.762	12.102	17.02%	0.761	12.203	17.71%
VI&TP	12	PLSR	0.763	13.385	16.08%	0.746	13.375	16.20%
		SVR	0.784	12.554	15.43%	0.776	12.221	15.59%
		RFR	0.791	12.531	15.51%	0.794	11.804	15.07%
VI&TP&TF	17	PLSR	0.840	11.606	14.07%	0.831	10.947	14.09%
		SVR	0.857	11.277	13.66%	0.854	10.200	13.07%
		RFR	0.872	10.108	12.54%	0.862	10.049	12.31%

## Data Availability

Not applicable.

## References

[1] Zhang N., Yang G., Pan Y., Yang X., Chen L., Zhao C. (2020). A Review of advanced technologies and development for hyperspectral-based plant disease detection in the past three decades. Remote Sens..

[2] Zhang J., Huang Y., Pu R., Gonzalez-Moreno P., Yuan L., Wu K., Huang W. (2019). Monitoring plant diseases and pests through remote sensing technology: A review. Comput. Electron. Agric..

[3] Feng W., Qi S., Heng Y., Zhou Y., Wu Y., Liu W., He L., Li X. (2017). Canopy vegetation indices from in situ hyperspectral data to assess plant water status of winter wheat under powdery mildew stress. Front. Plant Sci..

[4] Shi Y., Huang W., González-Moreno P., Luke B., Dong Y., Zheng Q., Ma H., Liu L. (2018). Wavelet-based rust spectral feature set (wrsfs): A novel spectral feature set based on continuous wavelet transformation for tracking progressive host–pathogen interaction of yellow rust on wheat. Remote Sens..

[5] Chen T., Zhang J., Chen Y., Wan S., Zhang L. (2019). Detection of peanut leaf spots disease using canopy hyperspectral reflectance. Comput. Electron. Agric..

[6] Franceschini M.H.D., Bartholomeus H., van Apeldoorn D.F., Suomalainen J., Kooistra L. (2019). Feasibility of unmanned aerial vehicle optical imagery for early detection and severity assessment of late blight in potato. Remote Sens..

[7] Mahlein A.K., Rumpf T., Welke P., Dehne H.W., Plümer L., Steiner U., Oerke E.C. (2013). Development of spectral indices for detecting and identifying plant diseases. Remote Sens. Environ..

[8] Feng W., Shen W., He L., Duan J., Guo B., LI Y., Wang C., Guo T. (2016). Improved remote sensing detection of wheat powdery mildew using dual-green vegetation indices. Precis. Agric..

[9] Naidu P.A., Perry E.M., Pierce F.J., Mekuria T. (2009). The potential of spectral reflectance technique for the detection of Grapevine leafroll associated virus-3 in two red-berried wine grape cultivars. Comput. Electron. Agric..

[10] Zhang J., Pu R., Wang J., Huang W., Yuan L., Luo J. (2012). Detecting powdery mildew of winter wheat using leaf level hyperspectral measurements. Comput. Electron. Agric..

[11] Tian L., Xue B., Wang Z., Li D., Yao X., Cao Q., Zhu Y., Cao W., Cheng T. (2021). Spectroscopic detection of rice leaf blast infection from asymptomatic to mild stages with integrated machine learning and feature selection. Remote Sens. Environ..

[12] Badnakhe M.R., Durbha S.S., Jagarlapudi A., Gade R.M. (2018). Evaluation of citrus gummosis disease dynamics and predictions with weather and inversion based leaf optical model. Comput. Electron. Agric..

[13] Ashourloo D., Aghighi H., Matkan A.A., Mobasheri M.R., Rad A.M. (2016). An investigation into machine learning regression techniques for the leaf rust disease detection using hyperspectral measurement. IEEE J. Sel. Top. Appl. Earth Obs. Remote Sens..

[14] Gu Q., Sheng L., Zhang T., Lu Y., Zhang Z., Zheng K., Hu H., Zhou H. (2019). Early detection of tomato spotted wilt virus infection in tobacco using the hyperspectral imaging technique and machine learning algorithms. Comput. Electron. Agric..

[15] Liu L., Dong Y., Huang W., Du X., Ma H. (2020). Monitoring wheat fusarium head blight using unmanned aerial vehicle hyperspectral imagery. Remote Sens..

[16] Chen P., Nicolas T., Wang J., Philippe V., Huang W., Li B. (2010). New index for crop canopy fresh biomass estimation. Spectrosc. Spectr. Anal..

[17] Zhao Y., Jing X., Huang W., Dong Y., Li C. (2019). Comparison of sun-induced chlorophyll fluorescence and reflectance data on estimating severity of wheat stripe rust. Spectrosc. Spectr. Anal..

[18] Zhang D., Chen G., Yin Y., Hu R., Gu C., Pan Z., Zhou X., Chen Y. (2020). Integrating spectral and image data to detect Fusarium head blight of wheat. Comput. Electron. Agric..

[19] Khan I.H., Liu H., Li W., Cao A., Wang X., Liu H., Cheng T., Tian Y., Zhu Y., Cao W. (2021). Early Detection of Powdery Mildew Disease and Accurate Quantification of Its Severity Using Hyperspectral Images in Wheat. Remote Sens..

[20] Zheng H., Cheng T., Zhou M., Li D., Yao X., Tian Y., Cao W., Zhu Y. (2019). Improved estimation of rice aboveground biomass combining textural and spectral analysis of UAV imagery. Precis. Agric..

[21] Al-Saddik H., Laybros A., Billiot B., Cointault F. (2018). Using image texture and spectral reflectance analysis to detect yellowness and esca in grapevines at leaf-level. Remote Sens..

[22] Guo A., Huang W., Dong Y., Ye H., Ma H., Liu B., Wu W., Ren Y., Ruan C., Geng Y. (2021). Wheat yellow rust detection using uav-based hyperspectral technology. Remote Sens..

[23] Khanal S., Fulton J., Shearer S. (2017). An overview of current and potential applications of thermal remote sensing in precision agriculture. Comput. Electron. Agric..

[24] Mahlein A.K., Alisaac E., Al Masri A., Behmann J., Dehne H.W., Oerke E.C. (2019). Comparison and combination of thermal, fluorescence, and hyperspectral imaging for monitoring fusarium head blight of wheat on spikelet scale. Sensors.

[25] Zarco-Tejada P.J., Camino C., Beck P.S.A., Calderon R., Hornero A., Hernández-Clemente R., Kattenborn T., Montes-Borrego M., Susca L., Morelli M. (2018). Previsual symptoms of Xylella fastidiosa infection revealed in spectral plant-trait alterations. Nat. Plants.

[26] Poblete T., Camino C., Beck P.S.A., Hornero A., Kattenborn T., Saponari M., Boscia D., Navas-Cortes J.A., Zarco-Tejada P.J. (2020). Detection of Xylella fastidiosa infection symptoms with airborne multispectral and thermal imagery: Assessing bandset reduction performance from hyperspectral analysis. ISPRS J. Photogramm. Remote Sens..

[27] Zhang C., Chen W., Sankaran S. (2019). High-throughput field phenotyping of ascochyta blight disease severity in chickpea. Crop Prot..

[28] Xie C., Yang C. (2020). A review on plant high-throughput phenotyping traits using UAV-based sensors. Comput. Electron. Agric..

[29] Zhang J., Pu R., Yuan L., Huang W., Nie C., Yang G. (2017). Integrating remotely sensed and meteorological observations to forecast wheat powdery mildew at a regional scale. IEEE J. Sel. Top. Appl. Earth Obs. Remote Sens..

[30] Maimaitijiang M., Sagan V., Sidike P., Harting S., Fritschi F.B. (2019). Soybean yield prediction from UAV using multimodal data fusion and deep learning. Remote Sens. Environ..

[31] (2015). Technical Specification on Remote Sensing Monitoring for Crop Diseases—Part 2: Wheat Powder Mildew.

[32] Zhang J., Cheng T., Guo W., Xu X., Qiao H., Xie Y., Ma X. (2021). Leaf area index estimation model for UAV image hyperspectral data based on wavelength variable selection and machine learning methods. Plant Methods.

[33] Haboudane D., Miller J.R., Pattey E., Zarco-Tejada P.J., Strachan I.B. (2004). Hyperspectral vegetation indices and novel algorithms for predicting green LAI of crop canopies: Modeling and validation in the context of precision agriculture. Remote Sens. Environ..

[34] Gamon J., Penuelas J., Field C. (1992). A narrow-waveband spectral index that tracks diurnal changes in photosynthetic efficiency. Remote Sens. Environ..

[35] Daughtry C.S.T., Walthall C.L., Kim M.S., Colstoun E.B., McMurtrey J.E. (2000). Estimating corn leaf chlorophyll concentration from leaf and canopy reflectance. Remote Sens. Environ..

[36] Haboudane D., Miller J.R., Tremblay N., Zarco-Tejada P.J., Dextraze L. (2002). Integrated narrow-band vegetation indices for prediction of crop chlorophyll content for application to precision agriculture. Remote Sens. Environ..

[37] Abdulridha J., Ampatzidis Y., Kakarla S.C., Roberts P. (2019). Detection of target spot and bacterial spot diseases in tomato using UAV-based and benchtop-based hyperspectral imaging techniques. Precis. Agric..

[38] Penuelas J., Frédéric B., Filella I. (1995). Semi-empirical indices to assess carotenoids/chlorophyll A ratio from leaf spectral reflectances. Photosynthetica.

[39] Gitelson A.A., Kaufman Y.J., Stark R., Rundquist D. (2002). Novel algorithms for remote estimation of vegetation fraction. Remote Sens. Environ..

[40] Roujean J.L., Broen F.M. (1995). Estimating PAR absorbed by vegetation from bidirectional reflectance measurements. Remote Sens. Environ..

[41] Gitelson A.A., Merzlyak M.N., Chivkunova O.B. (2010). Optical properties and nondestructive estimation of anthocyanin content in plant leaves. Photochem. Photobiol..

[42] Mirik M., Michels G.J., Kassymzhanova-Mirik S., Elliott N.C., Catana V., Jones D.B., Bowling R. (2006). Using digital image analysis and spectral reflectance data to quantify damage by greenbug (Hemitera: Aphididae) in winter wheat. Comput. Electron. Agric..

[43] Zarco-tejada P.J., Berjón A., López-lozano R., Miller J.R., Martín P., Cachorro V., González M.R., Frutos A.D. (2005). Assessing vineyard condition with hyperspectral indices: Leaf and canopy reflectance simulation in a row-structured discontinuous canopy. Remote Sens. Environ..

[44] Merzlyak M.N., Gitelson A.A., Chivkunova O.B., Rakitin V.Y. (1999). Non-destructive optical detection of pigment changes during leaf senescence and fruit ripening. Physiol. Plant..

[45] Filella I., Serrano L., Serra J., Peñuelas J. (1995). Evaluating wheat nitrogen status with canopy reflectance indices and discriminant analysis. Crop Sci..

[46] De Castro A., Ehsani R., Ploetz R., Crane J.H., Abdulridha J. (2015). Optimum spectral and geometric parameters for early detection of laurel wilt disease in avocado. Remote Sens. Environ..

[47] Gitelson A.A., Kaufman Y.J., Merzlyak M.N. (1996). Use of a green channel in remote sensing of global vegetation from EOS-MODIS. Remote Sens. Environ..

[48] Liu L., Huang W., Pu R., Wang J. (2014). Detection of internal leaf structure deterioration using a new spectral ratio index in the near-infrared shoulder region. J. Integr. Agric..

[49] Huete A.R. (1988). A soil-adjusted vegetation index (SAVI). Remote Sens. Environ..

[50] Haralick R.M., Shanmugam K., Dinstein I. (1973). Textural features for image classification. IEEE Trans. Cybern..

[51] Prasad S., Peddoju S.K., Ghosh D. (2016). Multi-resolution mobile vision system for plant leaf disease diagnosis. Signal Image Video Process..

[52] Li S., Yuan F., Ata-UI-Karim S.T., Zheng H., Cheng T., Liu X., Tian Y., Zhu Y., Cao W., Cao Q. (2019). Combining Color Indices and Textures of UAV-Based Digital Imagery for Rice LAI Estimation. Remote Sens..

[53] Guo J., Zhang J., Xiong S., Zhang Z., Wei Q., Zhang W., Feng W., Ma X. (2021). Hyperspectral assessment of leaf nitrogen accumulation for winter wheat using different regression modeling. Precis. Agric..

[54] Han J., Zhang Z., Cao J., Luo Y., Zhang L., Li Z., Zhang J. (2020). Prediction of winter wheat yield based on multi-source data and machine learning in China. Remote Sens..

[55] Breiman L. (2001). Random forest. Mach. Learn..

[56] Lu B., He Y. (2019). Evaluating empirical regression, machine learning, and radiative transfer modelling for estimating vegetation chlorophyll content using bi-seasonal hyperspectral images. Remote Sens..

[57] Graeff S., Link J., Claupein W. (2006). Identification of powdery mildew (*Erysiphe graminis* sp. *tritici*) and take-all disease (*Gaeumannomyces graminis* sp. *tritici*) in wheat (*Triticum aestivum* L.) by means of leaf reflectance measurements. Cent. Eur. J. Biol..

[58] Shi Y., Huang W., Zhou X. (2017). Evaluation of wavelet spectral features in pathological detection and discrimination of yellow rust and powdery mildew in winter wheat with hyperspectral reflectance data. J. Appl. Remote Sens..

[59] Zhao J., Fang Y., Chu G., Yan H., Hu L., Huang L. (2020). Identification of leaf-scale wheat powdery mildew (*Blumeria graminis* f. sp. *Tritici*) combining hyperspectral imaging and an SVM classifier. Plants.

[60] Rajendran D.K., Park E., Nagendran R., Hung N.B., Cho B.K., Kim K.H., Lee Y.H. (2016). Visual analysis for detection and quantification of pseudomonas cichorii disease severity in tomato plants. Plant Pathol. J..

[61] Xie C., He Y. (2016). Spectrum and image texture features analysis for early blight disease detection on eggplant leaves. Sensors.

[62] Guo A., Huang W., Ye H., Dong Y., Ma H., Ren Y., Ruan C. (2020). Identification of wheat yellow rust using spectral and texture features of hyperspectral images. Remote Sens..

[63] Cao W., Qiao Z., Gao Z., Lu S., Tian F. (2021). Use of unmanned aerial vehicle imagery and a hybrid algorithm combining a watershed algorithm and adaptive threshold segmentation to extract wheat lodging. Phys. Chem. Earth Parts A/B/C.

[64] Chandel A.K., Khot L.R., Yu L. (2021). crop vigor and yield characterization using high-resolution aerial multispectral and thermal infrared imaging technique. Comput. Electron. Agric..

[65] Francesconi S., Harfrouche A., Maesano M., Balestra G.M. (2021). UAV-based thermal, RGB imaging and gene expression analysis allowed detection of fusarium head blight and gave new insights into the physiological responses to the disease in durum wheat. Front. Plant Sci..

[66] Calderón A., Navas-Cortés J.A., Lucena C., Zarco-Tejada P.J. (2013). High-resolution airborne hyperspectral and thermal imagery for early detection of Verticillium wilt of olive using fluorescence, temperature and narrow-band spectral indices. Remote Sens. Environ..

[67] Han Z., Deng L. (2018). Application driven key wavelengths mining method for aflatoxin detection using hyperspectral data. Comput. Electron. Agric..

[68] Zheng Q., Huang W., Cui X., Shi Y., Liu L. (2018). New spectral index for detecting wheat yellow rust using sentinel-2 multispectral imagery. Sensors.

[69] Jiang X., Zhen J., Miao J., Zhao D., Wang J., Jia S. (2021). Assessing mangrove leaf traits under different pest and disease severity with hyperspectral imaging spectroscopy. Ecol. Indic..

[70] Zhang D., Chen G., Zhang H., Jing X., Gu C., Weng S., Wang Q., Chen Y. (2020). Integration of spectroscopy and image for identifying fusarium damage in wheat kernels using hyperspectral imaging. Spectrochim. Acta Part A.

[71] Meiforth J.J., Buddenbaum H., Hill J., Shepherd J. (2020). Monitoring of canopy stress symptoms in New Zealand kauri trees analysed with AISA hyperspectral data. Remote Sens..

[72] Burnett A.C., Anderson J., Davidson K.J., Ely K.S., Lamour J., Li Q., Morrison B.D., Yang D., Rogers A., Serbin S.P. (2021). A best-practice guide to predicting plant traits from leaf-level hyperspectral data using partial least squares regression. J. Exp. Bot..

